# AI can see you: Machiavellianism and extraversion are reflected in eye-movements

**DOI:** 10.1371/journal.pone.0308631

**Published:** 2024-08-28

**Authors:** Elina Tsigeman, Viktoria Zemliak, Maxim Likhanov, Kostas A. Papageorgiou, Yulia Kovas

**Affiliations:** 1 Laboratory for Social & Cognitive Informatics, HSE University, Saint-Petersburg, Russia; 2 University of Osnabrück, Osnabrück, Germany; 3 State Key Laboratory of Cognitive Neuroscience and Learning, Beijing Normal University, Beijing, China; 4 School of Psychology, Queen’s University Belfast, Belfast, United Kingdom; 5 Department of Psychology, Goldsmiths University of London, London, United Kingdom; COMSATS University Islamabad, PAKISTAN

## Abstract

**Introduction:**

Recent studies showed an association between personality traits and individual patterns of visual behaviour in laboratory and other settings. The current study extends previous research by measuring multiple personality traits in natural settings; and by comparing accuracy of prediction of multiple machine learning algorithms.

**Methods:**

Adolescent participants (N = 35) completed personality questionnaires (Big Five Inventory and Short Dark Triad Questionnaire) and visited an interactive museum while their eye movements were recorded with head-mounted eye tracking. To predict personality traits the eye-movement data was analysed using eight machine-learning methods: Random Forest, Adaboost, Naive Bayes, Support Vector Machine, Logistic Regression, k Nearest Neighbours, Decision Tree and a three-layer Perceptron.

**Results and discussion:**

Extracted eye movement features introduced to machine learning algorithms predicted personality traits with above 33% chance accuracy (34%–48%). This result is comparable to previous ecologically valid studies, but lower than in laboratory-based research. Better prediction was achieved for Machiavellianism and Extraversion compared to other traits (10 and 9 predictions above the chance level by different algorithms from different parts of the recording). Conscientiousness, Narcissism and Psychopathy were not reliably predicted from eye movements. These differences in predictability across traits might be explained by differential activation of different traits in different situations, such as new vs. familiar, exciting vs. boring, and complex vs. simple settings. In turn, different machine learning approaches seem to be better at capturing specific gaze patterns (e.g. saccades), associated with specific traits evoked by the situation. Further research is needed to gain better insights into trait-situation-algorithm interactions.

## 1 Introduction

The increasing use of personality assessment in research, clinical, occupational and educational settings requires easy-to-administer, reliable personality measures. Currently, researchers and practitioners rely predominantly on self-assessments (e.g. BFI [1]; SD3 [2]) or reports from friends and relatives [3, 4]. Despite all advantages (speed of testing, conceptual and analytical simplicity and inexpensiveness), this approach is limited by positive or negative bias, halo and social desirability effects [3]. Ipsative or forced-choice scales can overcome some of these biases but present challenges in scoring and interpretation [5]. Recently, analysis of digital traces (e.g., Facebook likes [4, 6]) and psychophysiological estimates, such as EEG (electroencephalographic) markers [7] and eye-tracking [8] were introduced to the field (see review [9]).

Eye-tracking is the non-invasive recording of gaze and reflects biological features of the human visual system, as well as typical behavioural patterns [10, 11]. This method is easy to implement compared to the more tedious and invasive EEG or the computationally intensive analysis of digital traces. Moreover, gaze features are stable over time and exhibit a high degree of individual uniqueness [12]. Head-mounted eye trackers also offer good ecological validity, as they allow gaze recording in real-life situations. In contrast, EEG method suffers from movement artefacts [13]; and digital traces are limited to online interactions (e.g. browsing social networks). Furthermore, the recording quality of eye-trackers is increasing and their cost is decreasing [14]–leading to their growing use in research, educational or clinical practice [15, 16]. For example, a recent study demonstrated that smartphone-based eye tracking technology has reached a level of accuracy suitable for use in both research and practical applications, further increasing this technology availability [17].

Recent studies have used eye-tracking to investigate associations between personality traits and gaze patterns [8, 10, 18–22]. For example, in one study individuals exhibited consistent visual behaviour (fixations, mean fixation duration and dwelling time) when presented with abstract animated stimuli that differed in several features (bright vs. soft colours; slow vs. jerky movements; [10]). This consistency was argued to be driven by personality traits (i.e. Big Five and behavioural inhibition/activation systems). Another study predicted future behavioural problems (e.g., Hyperactivity/Inattention) and surgency from eye movements recorded as early as 2 days after birth [23].

However, the mechanisms of the links between personality and gaze are poorly understood. Recent research suggested that personality and the visual system might share a common neural substrate. For example, in one fMRI study the personality-associated network overlapped with the visual system in the brain, specifically medial occipital cortex, lateral occipital cortex and fusiform gyrus areas [24]. Further, in accordance with *a trait congruency model* [25], affective processes may impact visual behaviour. For example, several studies showed that individuals diagnosed with affective disorders such as anxiety and depression or scoring high on Neuroticism tend to look at threatening and unpleasant stimuli longer than individuals without such diagnoses [26, 27], while people low on anxiety, depression and neuroticism show protective attentional biases, such as avoiding negative stimuli [28]. Further, recent research showed an association between scores on grandiose narcissism and variability in the number of eye fixations during cognitive task performance, supporting the link between personality and allocation of visual attention [29].

Previous research demonstrated that personality traits are linked to processing of social cues, prosocial behaviour and social strategies [30]; sensitivity to social threats [31] and attachment styles [32]. For example, one study showed that people with high charisma trait scores tended to gaze more frequently and for longer periods at people in a recorded scene at a busy supermarket [33]. Another study showed differences in gaze behaviour during processing of facial expressions between individuals with high vs. low extraversion scores [34].

At the same time, social signals can modulate gaze behaviour. According to recent findings, gaze behaviour serves both sensing and signalling functions during interaction and cooperation [35, 36]. Multiple studies show that mere feeling of social presence (true or false) affects gaze behaviour [37–39]. One study also showed that social stimuli (e.g. pictures of angry or happy faces) presented as briefly as for 50 milliseconds without conscious awareness were still able to affect gazing [40]. Previous research also highlighted that social presence can be inferred from eye-tracking: it is argued that the presence of eye-tracker makes people change their visual behaviour in accordance with social norms [41]. Moreover, research has found that social context moderates gaze behaviour irrespective of personality traits. For example, one study showed that participants, irrespective of their personality traits or social skills, largely avoided social gaze in an experimental waiting room scenario, with only 22% of fixations falling on face and body of another person [42].

Overall, existing studies highlights the complexity of the links among personality, gaze and social situations, calling for further research that examines greater number of traits and gaze features within one study. Previous studies that linked eye movements and personality studies have mainly focused on the Five-Factor personality model [10, 18–20, 22, 27, 43–47], curiosity [21, 48], BIS/BAS [8, 10, 49] or optimism [50], with only a few studies examining other traits, such as the Dark Triad [8, 51, 52]. Furthermore, most studies used a restricted number of gaze features such as dwelling time, number of fixations, and fixation duration [10, 49, 51], missing more subtle gaze features such as blink rate.

Machine learning (ML) approaches are increasingly being used to explore complex relationships of cognitive and neuroscience data such as EEG [53], MEG [54], fMRI [55, 56] and structural MRI [55]. For example, Berkovsky and collaborators [8] investigated the link between personality and eye movements by applying seven machine learning algorithms to predict 16 personality traits (Dark Triad, HEXACO and BIS/BAS scales) from ten eye movement features recorded during exposure to affective images and video stimuli. Results showed an average prediction accuracy of 85%.

Most previous research in this area has been conducted in laboratory settings [8, 11, 22, 52, 57]. Previous studies highlighted the profound differences in visual behaviour between the lab and the natural environment [57]. It is possible that data obtained in ecologically valid settings might bring new insights regarding the mechanisms of the link between personality and gaze behaviour [58]. For example, one study outside the laboratory predicted Big Five traits and curiosity from 207 gaze features recorded as participants walked across a university campus [59]. This study used *Random forest* machine learning algorithm that predicted all Big Five traits, except Openness, with an accuracy ranging from 37% to 48% (above 33% chance level). Accuracy of prediction was partially related to the task and environment at the time of recording. Specifically, prediction accuracy for personality traits was higher when eye movements were recorded on the way to/from the shop (.60 -.70) compared to those recorded during buying groceries at the shop (.39–63). These differences in prediction accuracy require further investigation and might be linked with a variety of factors, such as absence/presence of other people, purpose of activity, and other differences in circumstances.

In the current study, we extend previous research by predicting several personality traits from multiple eye movement features recorded in a natural setting as in [59] and by applying multiple machine learning algorithms to the same data as in [8]. Specifically, we aim to investigate: (a) predictability of the Big Five and the Dark Triad personality traits from eye movement features; (b) predictability of eye movements recorded during museum exploration vs. journey to/from museum; and (c) efficiency of different machine learning algorithms.

## 2 Materials and method

### 2.1 Participants

Thirty-five adolescents (24 males, 1 participant did not provide gender information; *M* age = 15.03; *SD* = 1.01; age range = 14–18 years) were recruited in 2018–2019. All participants were students in different schools in Russia and at the time of data collection attended extracurricular programmes at an educational centre. As part of their activities these students were invited to participate in the current study. Only participants with normal vision were recruited. Participants who wore eyeglasses or contact lenses were not included as they present challenges for capturing eye-movements with head-mounted eye-tracker. Due to technical problems and missing data, data from five participants were excluded. The final sample comprised 30 participants (20 males; *M* age = 14.09; *SD* = 0.99) with complete data.

Ethics Committee for Interdisciplinary Investigations, Tomsk State University (code of ethical approval: 16012018–5) approved the study. Participants’ parents’ or guardians’ written informed consent and participants’ assent were obtained.

### 2.2 Eye-tracking apparatus

Binocular gaze data were recorded with a head-mounted Tobii Pro Glasses 2 eye tracker at 50 Hz previously used in similar studies (see e.g. [49]). The tracker recorded gaze data together with a high-resolution video recording of participant’s visual scene.

### 2.3 Personality questionnaires

Two well-established personality measures were used to assess personality: the Big Five Inventory (BFI [1]) and the Short Dark Triad Questionnaire (SD3 [2]). The Russian adaptation of the Big Five Inventory [60] was used to assess five personality traits: Neuroticism, Extraversion, Openness to experience, Agreeableness, and Conscientiousness [1]. The questionnaire consists of 44 statements evaluated on a five-point Likert’s scale from 1 (Strongly disagree) to 5 (Strongly agree). To assess the Dark Triad traits (Machiavellianism, subclinical Narcissism, and subclinical Psychopathy) the Russian adaptation [61] of the Short Dark Triad questionnaire (SD3 [2]) was used. The questionnaire contains 27 statements evaluated on a five-point Likert scale from 1 (Strongly disagree) to 5 (Strongly agree). The reliability estimates for both measures obtained from a similar Russian adolescent sample were shown to be sufficient [62]. Overall, the scores are similar to those previously reported for Russian [62, 63] and other samples [64, 65].

### 2.4 Procedure

Participants first filled in computerised personality questionnaires. Then, participants put on a head-mounted eye-tracker and a one-point calibration was performed. The experimenter led participants down a hallway to the interactive museum where exhibits of modern gadgets and technologies (e.g., fitness tracking bracelets, VR and AR) and explanations of their use were presented. In experimental condition, participants explored the museum for 10 minutes, interacting with exhibits of their choice, for example by clicking buttons and moving levers (The Museum part). Afterwards, the experimenter led the participants back to the lab via the same hallway. The walk to and back from the museum served as a control condition (The Way part). During the walk, participants received no instructions and were free to behave naturally, look around and talk to the experimenter. In both the experimental and control conditions, participants could encounter other people in the building.

### 2.5 Data processing and analyses

On average, 15.47 minutes (*SD* = 1.87) of eye-tracking data were collected per participant. Recording took an average of 10.75 minutes (*SD* = 3.32) within the museum and 4.86 minutes (*SD* = 0.94) travelling from the lab to the museum and back.

The data was analysed using the Python 3.7 programming language (RRID: SCR_008394) following the procedures used in Hoppe and collaborators [59]. We adapted an existing Python 2 code to Python 3 to fit to the eye-tracking device used in the current study.

As suggested in Hoppe and collaborators [59], the scores for each personality trait were divided into three ranges (low, medium and high) so that the number of cases in each group was approximately equal (see S2 Table for the number of participants for each range).

Overall 207 gaze features were extracted, using the procedure proposed by Hoppe and collaborators [59]. The recording was divided into two parts: museum exploration vs. journey to/from museum. Analyses were conducted on Museum part, Way part and combined full recording (Museum + Way).

For each personality trait, we trained eight ML classification algorithms: *Random forest* (*RM*, similar to Hoppe and collaborators [59]; *Adaboost*, *Naive Bayes (NB)*, *Support Vector Machine (SVM)*, *Logistic Regression* (*LR*), *k Nearest Neighbours (kNN)* and *Decision Tree (DT)–*algorithms that were used in Berkovsky and collaborators [8]; and a *three-layer Perceptron* method with *ReLu* activation functions between the hidden layers [66],–similar to Chen and collaborators [43]. For the neural network, we used an implementation of the multi-layer perceptron from *the scikit-learn* package, with three hidden layers of sizes 256, 128 and 64. The values of other parameters we kept default: Rectified linear unit (*ReLu*) was used as an activation function in all hidden layers, and *Adam* optimizer was used for optimization.

We used the F-measure (the harmonic mean of precision and recall calculated on the test set) to evaluate the performance of the algorithms. We compared the baseline data and the results of eight algorithms obtained during cross-validation across all features under three conditions: (1) on the whole recording; (2) on the way to the museum and back to the lab, and (3) inside the museum. We consider a feature to be successfully predicted only if at least two algorithms predicted it, and we refer to it as *consistent prediction*. For details on Feature Extraction, Classification Procedure and Classifiers evaluation see Supporting information.

## 3 Results

The data on all personality traits was normally distributed and compatible with previously reported data from Russian adolescents [62, 63]. Internal consistency was excellent for Big Five (>.81) and good-to-excellent for Dark Triad (>.69). Descriptive statistics on the personality traits can be found in S1 Table.

### 3.1 Classification results

**Fig 1:**
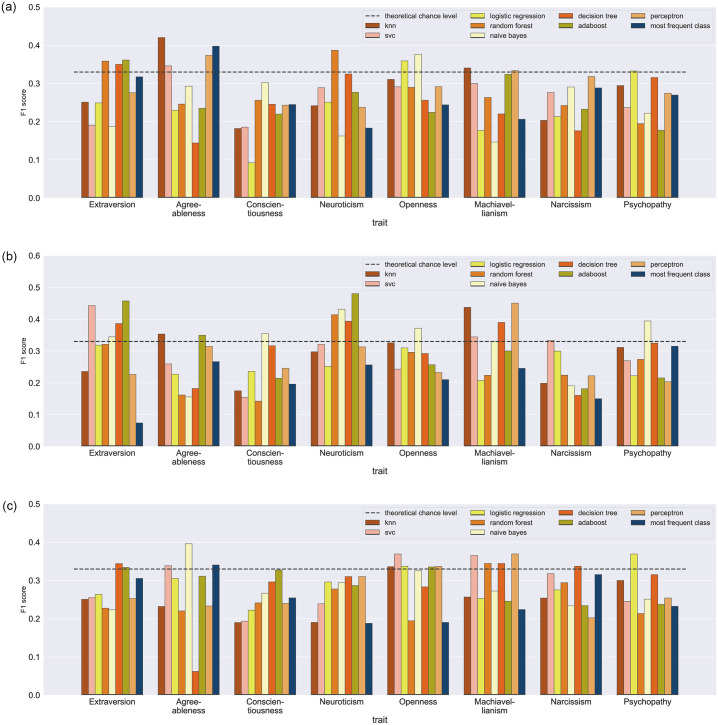
Mean F1 scores for the eight classifiers predicting personality traits and the baselines of the most frequently occurring class. A. Museum + Way, B. Way, C. Museum. *Note* All results were obtained using a cross-validation procedure such that only predictions for participants not included in training set were used for evaluation. The dashed line shows the theoretical chance level (33%) for a classifier that randomly picks one personality score range for each participant.

Fig 1 shows the mean F1 score for the algorithms trained on the whole recording (Panel A), the Way part (Panel B), and the Museum part (Panel C) of the data, as well as two baselines for each trait (the random class of three possible score ranges and the most frequent class from the training set on the test set.). The F1 scores for each trait from all the classifiers are available in S3 Table.

For the Way + Museum recording (Fig 1 Panel A), consistent prediction was achieved for Extraversion, Agreeableness and Openness. Only one prediction above the chance level was obtained for Neuroticism, Machiavellianism and Psychopathy. No prediction above chance was shown for Narcissism and Conscientiousness. The highest F1 value was found for Agreeableness using the *KNN* algorithm (.42).

For the Way part (Fig 1 Panel B) consistent prediction was achieved for Neuroticism, Extraversion, Machiavellianism, and Agreeableness. Openness, Conscientiousness, Narcissism, and Psychopathy were predicted above chance by only one algorithm. The highest scores were obtained for Neuroticism (.48, *Adaboost*), Extraversion (.45, *Adaboost*) and Machiavellianism (.45, *Three-layer Perceptron*).

For Museum part (Fig 1 Panel C), the consistent prediction was reached for Extraversion, Openness, Agreeableness and Machiavellianism. Narcissism and Psychopathy were predicted above chance by only one algorithm. No prediction above chance was shown for Neuroticism and Conscientiousness.

Overall, out of the three analyses groups, the Way part yielded the highest and the biggest number of predictions (18 predictions above the chance level, F1_max_ = .48), compared to Museum (15 predictions, F1_max_ = .39) and Way + Museum (12 predictions, F1_max_ = .42) recording. Of 8 investigated traits, the lest predictable traits were Conscientiousness and Narcissism.

The most successful algorithm was *NB* (8 successful predictions). *Perceptron*, *KNN*, *Adaboost*, *SVM* and *DT* algorithms provided 5–7 successful predictions. The least successful algorithms were *LR* and *RF* with only 4 predictions above chance level.

### 3.2 Classification by low/medium/high range of the traits

We also conducted additional analysis to test whether the accuracy of prediction of three personality trait differs as per trait level (low, medium and high) following approach from [44]. No clear pattern of differences emerged with some traits predicted better at high levels (e.g. Openness and Agreeableness), whereas some predicted better at low (Machiavellianism) or medium levels (e.g. Machiavellianism, Psychopathy or Narcissism). All DT traits show poor predictability of high score range. The full results are presented in S4 Table.

## 4 Discussion

In the current study we applied eight machine learning algorithms to the eye movement features recorded in two natural setting conditions to predict several personality traits. We predicted four traits out of Big Five and one trait out of Dark Triad from participants’ eye movements with 34–48% precision (above 33% theoretical chance level).

We evaluated our findings in the context of previous research. Prediction accuracy in the current study was comparable to that obtained in Hoppe and collaborators [59], who also used an ecologically valid setting. However, it was lower as compared to the predictability of 70%-90% reached in some laboratory experiments [8, 22, 43, 45]. More research is needed to understand the source of differences in results. For example, the differences may be due setting: unstructured natural settings vs. highly controlled laboratory settings [57]. In addition, the paradigm differed across the studies. For example, in Berkovsky and collaborators [8] emotionally or socially loaded pictures and videos were used during the recording as stimuli.

The most predictable traits in the current experiment were Machiavellianism and Extraversion (10 and 9 predictions above the chance level by different algorithms from different parts of the recording). High predictability of Machiavellianism from eye movements was found in previous research [8]. This link might be explained by experimental situation that required high impulse control over gaze from participants as they knew their gaze was recorded; and according to recent theoretical considerations, Machiavellianism is associated with elevated levels of impulse control and impression management [67]. Interestingly, Psychopathy had lower predictability than Machiavellianism in the current (only 3 predictions above the chance level) and the previous studies [8]. This pattern of results suggest that eye tracking can tap into some distinct features of Machiavellianism and Psychopathy that are differentially activated in different contexts.

Previous research also showed robust links between Extraversion and gaze behaviour. For example, Extraversion was linked to visual attention [68], face recognition [69, 70], saccade inhibition [71] and information seeking [20]. The links between Extraversion and gaze might appear because domains of Extraversion (e.g. interest in external stimuli and impression seeking) are reflected in gazing patterns such as shorter dwelling time and higher number of shorter fixations [10].

In the current study predictability of personality traits was overall more consistent for the Way part of the recording compared with the Museum part and combined recording. This finding is in line with previous studies that highlight context-dependant variability in association between personality traits and eye movements [20, 43, 59]. Specifically, on the Way participants were accompanied by an experimenter and may have seen other people while in the museum there were left alone to explore the exhibition. This differences may have led to differences in social cues processing and social strategies that were previously shown to be closely linked to personality [30]. This might explain why Openness, Neuroticism and Agreeableness were successfully predicted 5–8 times in the current study and in the experiments with recommendation interfaces [43], while they did not arise in the experiment with social network browsing task, with no direct interactions with other people [44].

Moreover, in accordance with *Trait Activation Theory* [72], traits need specific environments to be expressed. For example, the current study showed low predictability of Conscientiousness—a trait that is linked to being efficient/careless and organized/disorganized [73]. This might stem from the absence of a more specific task that could draw out differences in Conscientiousness (e.g. assessment element at the end). Laboratory studies showed prediction accuracy of.40 -.70 for Conscientiousness using such tasks as information seeking [20], social network browsing [44] and decision making [43].

There was small variation in the performance of the algorithms on different data parts. The best classification algorithm for predictions on the complete recordings was *NB*, which showed eight above the chance predictions of different traits. This result is in line with recent studies, where *NB* also showed the highest accuracy [8, 52]. Several other algorithms (*SVM*, *DT*, *Adaboost*, *KNN*, and *three-layer Perceptron)* also showed above chance predictions (5–7).

For the Way data, *Adaboost* achieved the best accuracy of 48%. For the Museum data, the most efficient model was *NB* with 39% of accuracy. For the whole recording, *KNN* showed 42% of accuracy. These differences can be due to the differences in distributions of the Way and Museum data. For instance, tree-based algorithms such as *DT*, *RF*, *three-layer Perceptron*, and *AdaBoost* can handle non-linear data, because they build a tree-like model of consequent decisions [74]. In contrast, the *LR* is a linear algorithm that builds an optimal boundary between the samples of different classes [74]. The links between personality and eye-movements can be linear or non-linear depending on environment. For example, an extraverted person might be more likely to look at the researcher on the way to museum and feel particularly bored in the museum.

The current study has several practical implications. First, this study supports the feasibility of measuring personality traits using eye-tracking in naturalistic settings, providing more ecologically valid insights into individual differences. Second, the results of the comparisons of machine learning algorithms provide new information for development of more reliable predictive models for assessment of different personality traits in different contexts. Increased accuracy in prediction of personality traits using eye movement data will lead to new opportunities in education, human resources and clinical practice, where information about personality can improve effectiveness of interventions and decision making.

## 5 Limitations and future directions

The current study has several limitations. First, the sample included only adolescent participants who might not yet have developed stable personalities [75, 76]. Second, the free-viewing task, used to enhance ecological validity, complicated the interpretation of the results due to high heterogeneity of participants’ behaviour [11, 77]. Recent findings indicate that free-viewing task captures both conscious and unconscious eye movements, and therefore future research must also include field restriction tasks that help to disentangle the relationship between personality traits and eye movements [78]. Third, the small sample (though comparable to previous studies, e.g. [48, 49, 51]) precluded further analyses, such as exploring gaze-personality associations in males and females [12, 79].

To conclude, the current study provides further support for the association between eye movements and personality. Further research is needed to disentangle the influence of different conditions and environments, such as specific trait-evoking environments; entertaining or boring visual scenes; new or familiar situations; potential differences in lighting across the environments; time spent in different environments; and objects chosen for exploration. In addition, the current study indicates that some ML methods are more efficient in particular environments and/or tasks, and in detection of particular eye-movement features that are linked to personality.

Further research on the links between personality and visual perception and further advancements in ML algorithms may eventually allow developing a “real-world” test battery, where personality is assessed through participants interacting with different environments. This personality assessment might include more specific facets of personality, such as Excitement Seeking, Positive Emotions, Warmth, Gregariousness, Assertiveness, and Activity instead of general Extraversion factor [80].

## Supporting information

S1 File Feature extraction. (DOCX)

S2 File Classification procedure. (DOCX)

S3 File Classifiers evaluation. (DOCX)

S1 Table Descriptive statistics for personality traits. (DOCX)

S2 Table The number of participants in each personality score range. (DOCX)

S3 Table Classifiers’ performance for all traits. (DOCX)

S4 Table Classifiers’ performance by trait category. (DOCX)
