# Supplementary material for: AI can see you: Machiavellianism and extraversion are reflected in eye-movements
Source: PLoS One. 2024 Aug 28;19(8):e0308631. doi: 10.1371/journal.pone.0308631 (PMC11355565; doi:10.1371/journal.pone.0308631)
Supplement: S1 File — (DOCX) [file pone.0308631.s001.docx]

## **1 Feature Extraction**

To extract the features from raw eye-tracking data, the sliding window approach with 50% overlap was applied (Bulling et al., 2011). A feature vector of 207 dimensions was generated for each time window. The features included:

(1) statistics of raw eye-tracking data (e.g. mean, minimum, and maximum gaze coordinates);

(2) 64 cells of the heatmap, built from the raw gaze data;

(3) common statistics over most basic eye movements and events, such as saccades, fixations, and blinks, e.g. the saccades’ direction and the mean fixation duration. A built-in velocity-threshold algorithm (Anneli Olsen, 2012) with a threshold equal to 30 degrees per second and a minimum duration threshold of 60 ms was used for fixation detection. The adjacent fixations were merged if the time between them was less than 75 ms or if the angle was smaller than 0.5 degrees. The pupil size was also detected and used in a feature vector;

(4) various statistics over n-grams, i.e. series of gaze events representing the temporal course of eye movements. N refers to the number of saccades and fixations in a single time series and varies from 1 to 4. The statistics over n-grams included their relative quantity, the least/most frequent n-gram across the dataset, etc.

The vector of 207 features over each time window was used as a separate sample for classification. Thus, depending on time window size, the data from a single participant was transformed into multiple train, test and validation samples: from 3 to 415. Classification features were extracted from each time window data independently. That is, the overall training set for a single classifier could include from 100 to 12000 samples. This approach allowed to overcome the small amount of training data: if features had been calculated on the averaged data from a single participant, then the resulting training set size would have been no greater than the total number of participants.

The optimal window size for classification was selected through the nested cross-validation procedure. This procedure requires cycling through the whole set of participants in an outer and inner loop. In inner loop, the data is divided into train and validation sets. The classifiers inside of this inner loop are trained on various window sizes and subsets of features and evaluated on the validation set. Thus, the optimal window size and set of features are selected. In outer loop, the classifier with the selected features is evaluated on the separate test set.

All training, validation, and test data were standardized before the classification procedures. That is, the standard scaler was first fit to the training data and then applied to both training and testing data. This resulted in a zero mean and a standard deviation of one for each feature of 207 at each time window.
