# Supplementary material for: AI can see you: Machiavellianism and extraversion are reflected in eye-movements
Source: PLoS One. 2024 Aug 28;19(8):e0308631. doi: 10.1371/journal.pone.0308631 (PMC11355565; doi:10.1371/journal.pone.0308631)
Supplement: S2 File — (DOCX) [file pone.0308631.s002.docx]

## **2 Classification Procedure**

A similar procedure of classification as in Hoppe et al. (2018), and Berkovsky et al. (2019) was used. We trained eight algorithms for each personality trait independently, to evaluate: a) the predictability of various personality traits, i.e. how well each personality trait can be predicted from the eye-tracking data; b) the performance of each classifier, i.e. which methods of classification predict personality traits better.

All algorithms were implemented with the use of the *scikit-learn* package.

To train algorithms and effectively evaluate their performance, the nested cross-validation procedure was applied.

First, all participants were randomly split into six groups. At each iteration, the algorithms were trained on the data of five groups, while their performance was evaluated on the last testing group. Second, during the training stage all data from five groups were again split into three parts. For several iterations, two of them were used for re-training models with various features and parameter sets, while the last one was used for validating results. At this stage, the optimal subset of features and classifier parameters were selected. Finally, we used these features and parameters to train the algorithms on each of the six training sets and evaluated their performance on corresponding testing sets for each feature and classifier independently. We averaged the results across all testing sets, to get the final performance scores. Since the algorithm is trained, validated, and tested on various subsets of data, the nested cross-validation procedure helps to reduce the possibility of overfitting (Stone, 1974).

In this study we used both deterministic (*LR*, *SVM*, *Naive Bayes*, *kNN*) and non-deterministic (*RF*, *DT*, *AdaBoost*, and Perceptron) machine learning algorithms. To control for different results of non-deterministic algorithms when run with different initial random states, we repeated the entire nested-cross validation procedure 50 times with different random states and averaged their performance. Each of the other algorithms was run through the cross-validation only once.
