# Supplementary material for: AI can see you: Machiavellianism and extraversion are reflected in eye-movements
Source: PLoS One. 2024 Aug 28;19(8):e0308631. doi: 10.1371/journal.pone.0308631 (PMC11355565; doi:10.1371/journal.pone.0308631)
Supplement: S3 File — (DOCX) [file pone.0308631.s003.docx]

## **3 Classifiers Evaluation**

A similar procedure of the classifiers' performance evaluation as in Hoppe and collaborators (Hoppe et al., 2018) was conducted. To assess the prediction for a participant, the majority voting procedure was applied. Thus, the most frequent value (0 or 1) across all of the participant’s time windows predictions was considered a prediction for a single participant.

We finally calculated the F-measure based on the set of these predictions. The F-measure is the harmonic mean of two other metrics: Precision and Recall. Precision is the ratio of correctly predicted personality scores to all samples where this score was predicted. Recall refers to the number of samples with a correctly predicted score divided by the number of samples with this score in the test set.

To retrieve the final F-score for a given feature, precision and recall are first calculated for each of the three score ranges separately, and then F-scores are calculated upon them and averaged across the ranges. The final performance of classifiers was compared against two baselines, i.e. the performance of two rule-based classifiers making trivial predictions: (a) predicting the random class of three possible score ranges; (b) predicting the most frequent class from the training set on the test set.

The interpretation of the feature importance that was done in Hoppe et al. (2018) has been omitted in the present study, because different classification algorithms use different methods of calculating the feature importance, and quantify this importance differently. For example, *SVM* *with a linear kernel* calculates feature importance as the weight of the corresponding model coefficient. These coefficients represent the coordinates of the vector, which is orthogonal to a hyperplane dividing the classes. As for the *SVM with a radial kernel*, the direct feature importance calculation is impossible. In the *Decision Tree*, the feature importance implemented in the used *scikit-learn* package shows the average gain of homogeneity (purity) by splits of a given feature (Documentation: <https://scikit-learn.org/>). In the ensemble *Random Forest* classification algorithm, which is essentially a bunch of multiple decision trees, the feature importance is calculated with use of the same purity metric. However, even the documentation states that the calculation of feature importance can be misleading for features with many unique values. *AdaBoost* is another ensemble method based on decision trees, hence it utilizes the same method for computing the feature importance and suffers from the same vulnerabilities. The *Logistic Regression* provides the coefficients for the input features, which can be used to roughly estimate the feature importance. Some other methods, such as *Naïve Bayes classifier*, *KNN* or a *Multi-layer Perceptron*, do not have the specific intrinsic methods for computing the feature importance. In this case, more universal methods for calculating the feature importance could be used – e.g. the permutation importance. However, if we calculate the feature importance differently for different models, this makes it difficult to compare and interpret them. The feature importance is calculated separately and sometimes differently for each classifier model, and thus rather reflects the importance of the feature for a particular model than its intrinsic predictive value.
