# Supplementary material for: AI can see you: Machiavellianism and extraversion are reflected in eye-movements
Source: PLoS One. 2024 Aug 28;19(8):e0308631. doi: 10.1371/journal.pone.0308631 (PMC11355565; doi:10.1371/journal.pone.0308631)
Supplement: S1 Table — (DOCX) [file pone.0308631.s004.docx]

# **Table S1. Descriptive Statistics for personality traits**

| **Table S1.** Descriptive Statistics for personality traits | | | | | | | | |
| --- | --- | --- | --- | --- | --- | --- | --- | --- |
|  | **Min** | **Max** | **Mean (SD)** | **Skewness** | **Kurtosis** | **Cronbach’s α** | **McDonald's ω** |  |
| **Neuroticism** | 1.13 | 4.38 | 2.88 (0.8) | -0.32 | -0.28 | .811 | .813 |  |
| **Extraversion** | 1.25 | 4.63 | 3.39 (0.78) | -0.55 | 0.29 | .812 | .815 |  |
| **Openness** | 2.5 | 4.9 | 3.71 (0.72) | 0.32 | -1.37 | .816 | .828 |  |
| **Agreeableness** | 1.67 | 4.67 | 3.47 (0.7) | -0.56 | 0.06 | .823 | .834 |  |
| **Conscientiousness** | 2.33 | 4.78 | 3.4 (0.66) | 0.61 | -0.5 | .813 | .83 |  |
| **Machiavellianism** | 2 | 4.56 | 3.34 (0.59) | 0 | -0.01 | .708 | .724 |  |
| **Narcissism** | 2 | 4.67 | 3.03 (0.59) | 0.18 | 0.48 | .702 | .742 |  |
| **Psychopathy** | 1.22 | 3.78 | 2.23 (0.57) | 0.51 | 0.47 | .697 | .723 |  |
|  | | | | | | | | |
