# Supplementary material for: AI can see you: Machiavellianism and extraversion are reflected in eye-movements
Source: PLoS One. 2024 Aug 28;19(8):e0308631. doi: 10.1371/journal.pone.0308631 (PMC11355565; doi:10.1371/journal.pone.0308631)
Supplement: S2 Table — (DOCX) [file pone.0308631.s005.docx]

# **Table S2. The number of participants in each personality score range**

| **Table S2.** The number of participants in each personality score range | | | | | | | | |
| --- | --- | --- | --- | --- | --- | --- | --- | --- |
|  | **Neuroticism** | **Extraversion** | **Openness** | **Agreeableness** | **Conscientiousness** | **Machiavellianism** | **Narcissism** | **Psychopathy** |
| Low | 11 | 11 | 11 | 9 | 11 | 10 | 11 | 7 |
| Medium | 13 | 11 | 11 | 13 | 13 | 15 | 12 | 17 |
| High | 10 | 12 | 12 | 12 | 10 | 9 | 12 | 10 |
|  | | | | | | | | |
