# Supplementary material for: AI can see you: Machiavellianism and extraversion are reflected in eye-movements
Source: PLoS One. 2024 Aug 28;19(8):e0308631. doi: 10.1371/journal.pone.0308631 (PMC11355565; doi:10.1371/journal.pone.0308631)
Supplement: S3 Table — (DOCX) [file pone.0308631.s006.docx]

# **Table S3. Classifiers’ performance for all traits**

| **Table S3.** Classifiers’ performance for all traits | | | | | | |
| --- | --- | --- | --- | --- | --- | --- |
|  | **Whole recording** | | **Way** | | **Museum** | |
| **Trait** | **Algorithm** | **F1** | **Algorithm** | **F1** | **Algorithm** | **F1** |
| **Neuroticism** | Naive bayes | 0.162 | Logistic regression | 0.252 | K-nearest neighbour | 0.19 |
|  | Three-layer Perceptron | 0.237 | K-nearest neighbour | 0.298 | Support Vector Machine | 0.239 |
|  | K-nearest neighbour | 0.242 | Three-layer Perceptron | 0.313 | Random forest | 0.278 |
|  | Logistic regression | 0.251 | Support Vector Machine | 0.321 | Adaboost | 0.287 |
|  | Adaboost | 0.277 | **Decision tree** | **0.394** | Naive bayes | 0.294 |
|  | Support Vector Machine | 0.289 | **Random forest** | **0.414** | Logistic regression | 0.296 |
|  | Decision tree | 0.325 | **Naive bayes** | **0.431** | Decision tree | 0.31 |
|  | **Random forest** | **0.387** | **Adaboost** | **0.48** | Three-layer Perceptron | 0.31 |
| **Extraversion** | Naive bayes | 0.187 | Three-layer Perceptron | 0.226 | Naive bayes | 0.223 |
|  | Support Vector Machine | 0.191 | K-nearest neighbour | 0.236 | Random forest | 0.227 |
|  | Logistic regression | 0.249 | Logistic regression | 0.318 | K-nearest neighbour | 0.251 |
|  | K-nearest neighbour | 0.251 | Random forest | 0.321 | Three-layer Perceptron | 0.253 |
|  | Three-layer Perceptron | 0.276 | **Naive bayes** | **0.344** | Support Vector Machine | 0.255 |
|  | **Decision tree** | **0.35** | **Decision tree** | **0.386** | Logistic regression | 0.264 |
|  | **Random forest** | **0.359** | **Support Vector Machine** | **0.443** | **Adaboost** | **0.334** |
|  | **Adaboost** | **0.361** | **Adaboost** | **0.457** | **Decision tree** | **0.344** |
| **Openness** | Adaboost | 0.224 | Three-layer Perceptron | 0.232 | Random forest | 0.195 |
|  | Decision tree | 0.256 | Support Vector Machine | 0.243 | Decision tree | 0.283 |
|  | Random forest | 0.29 | Adaboost | 0.257 | Naive bayes | 0.325 |
|  | Support Vector Machine | 0.291 | Decision tree | 0.292 | **Adaboost** | **0.335** |
|  | Three-layer Perceptron | 0.292 | Random forest | 0.296 | **K-nearest neighbour** | **0.336** |
|  | K-nearest neighbour | 0.311 | Logistic regression | 0.31 | **Three-layer Perceptron** | **0.336** |
|  | **Logistic regression** | **0.359** | K-nearest neighbour | 0.326 | **Logistic regression** | **0.337** |
|  | **Naive bayes** | **0.376** | **Naive bayes** | **0.372** | **Support Vector Machine** | **0.369** |
| **Agreeableness** | Decision tree | 0.144 | Naive bayes | 0.156 | Decision tree | 0.062 |
|  | Logistic regression | 0.23 | Random forest | 0.162 | Random forest | 0.22 |
|  | Adaboost | 0.235 | Decision tree | 0.182 | K-nearest neighbour | 0.232 |
|  | Random forest | 0.246 | Logistic regression | 0.227 | Three-layer Perceptron | 0.233 |
|  | Naive bayes | 0.293 | Support Vector Machine | 0.259 | Logistic regression | 0.305 |
|  | **Support Vector Machine** | **0.346** | Three-layer Perceptron | 0.314 | Adaboost | 0.311 |
|  | **Three-layer Perceptron** | **0.374** | **Adaboost** | **0.35** | **Support Vector Machine** | **0.339** |
|  | **K-nearest neighbour** | **0.42** | **K-nearest neighbour** | **0.353** | **Naive bayes** | **0.396** |
| **Conscientiousness** | Logistic regression | 0.092 | Random forest | 0.142 | K-nearest neighbour | 0.19 |
|  | K-nearest neighbour | 0.182 | Support Vector Machine | 0.155 | Support Vector Machine | 0.194 |
|  | Support Vector Machine | 0.186 | K-nearest neighbour | 0.175 | Logistic regression | 0.222 |
|  | Adaboost | 0.22 | Adaboost | 0.214 | Three-layer Perceptron | 0.24 |
|  | Three-layer Perceptron | 0.243 | Logistic regression | 0.236 | Random forest | 0.242 |
|  | Decision tree | 0.246 | Three-layer Perceptron | 0.246 | Naive bayes | 0.267 |
|  | Random forest | 0.256 | Decision tree | 0.316 | Decision tree | 0.296 |
|  | Naive bayes | 0.302 | **Naive bayes** | **0.355** | Adaboost | 0.327 |
| **Machiavellianism** | Naive bayes | 0.147 | Logistic regression | 0.207 | Adaboost | 0.246 |
|  | Logistic regression | 0.177 | Random forest | 0.224 | Logistic regression | 0.253 |
|  | Decision tree | 0.22 | Adaboost | 0.3 | K-nearest neighbour | 0.257 |
|  | Random forest | 0.263 | **Naive bayes** | **0.331** | Naive bayes | 0.272 |
|  | Support Vector Machine | 0.3 | **Support Vector Machine** | **0.344** | **Decision tree** | **0.344** |
|  | Adaboost | 0.324 | **Decision tree** | **0.39** | **Random forest** | **0.344** |
|  | **Three-layer Perceptron** | **0.334** | **K-nearest neighbour** | **0.437** | **Support Vector Machine** | **0.365** |
|  | **K-nearest neighbour** | **0.341** | **Three-layer Perceptron** | **0.45** | **Three-layer Perceptron** | **0.37** |
| **Narcissism** | Decision tree | 0.176 | Decision tree | 0.16 | Three-layer Perceptron | 0.202 |
|  | K-nearest neighbour | 0.204 | Adaboost | 0.181 | Naive bayes | 0.234 |
|  | Logistic regression | 0.213 | Naive bayes | 0.191 | Adaboost | 0.234 |
|  | Adaboost | 0.233 | K-nearest neighbour | 0.199 | K-nearest neighbour | 0.254 |
|  | Random forest | 0.242 | Three-layer Perceptron | 0.222 | Logistic regression | 0.275 |
|  | Support Vector Machine | 0.276 | Random forest | 0.224 | Random forest | 0.294 |
|  | Naive bayes | 0.291 | Logistic regression | 0.3 | Support Vector Machine | 0.318 |
|  | Three-layer Perceptron | 0.318 | **Support Vector Machine** | **0.334** | **Decision tree** | **0.337** |
| **Psychopathy** | Adaboost | 0.177 | Three-layer Perceptron | 0.204 | Random forest | 0.213 |
|  | Random forest | 0.195 | Adaboost | 0.215 | Adaboost | 0.238 |
|  | Naive bayes | 0.222 | Logistic regression | 0.222 | Support Vector Machine | 0.245 |
|  | Support Vector Machine | 0.237 | Support Vector Machine | 0.269 | Naive bayes | 0.251 |
|  | Three-layer Perceptron | 0.274 | Random forest | 0.274 | Three-layer Perceptron | 0.254 |
|  | K-nearest neighbour | 0.295 | K-nearest neighbour | 0.311 | K-nearest neighbour | 0.3 |
|  | Decision tree | 0.316 | Decision tree | 0.325 | Decision tree | 0.315 |
|  | **Logistic regression** | **0.333** | **Naive bayes** | **0.394** | **Logistic regression** | **0.369** |
| Note: the predictions above the chance level of 33% are in bold. | | | | | | |
