# Supplementary material for: AI can see you: Machiavellianism and extraversion are reflected in eye-movements
Source: PLoS One. 2024 Aug 28;19(8):e0308631. doi: 10.1371/journal.pone.0308631 (PMC11355565; doi:10.1371/journal.pone.0308631)
Supplement: S4 Table — (DOCX) [file pone.0308631.s007.docx]

# **Table S4. Classifiers’ performance by trait category**

| **Table S4.** Classifiers’ performance by trait category | | | | | | | | | | | | |
| --- | --- | --- | --- | --- | --- | --- | --- | --- | --- | --- | --- | --- |
|  | **Whole recording** | | | | **Way** | | | | **Museum** | | | |
| **Trait** | **Algorithm** | **F1 low class** | **F1 medium class** | **F1 high class** | **Algorithm** | **F1 low class** | **F1 medium class** | **F1 high class** | **Algorithm** | **F1 low class** | **F1 medium class** | **F1 high class** |
| **Neuroticism** | Naive bayes | 0.181 | 0.21 | 0.095 | Logistic regression | 0.222 | **0.333** | 0.2 | K-nearest neighbour | 0.133 | **0.437** | 0 |
|  | Three-layer Perceptron | 0.21 | **0.385** | 0.118 | K-nearest neighbour | 0.143 | **0.417** | **0.333** | Support Vector Machine | 0.235 | **0.483** | 0 |
|  | K-nearest neighbour | 0.125 | **0.483** | 0.118 | Three-layer Perceptron | 0.3 | **0.417** | 0.222 | Random forest | 0.316 | **0.385** | 0.133 |
|  | Logistic regression | 0.235 | 0.308 | 0.21 | Support Vector Machine | 0.3 | **0.364** | 0.3 | Adaboost | 0.21 | **0.4** | 0.25 |
|  | Adaboost | 0.21 | **0.37** | 0.25 | Decision tree | 0.222 | **0.37** | **0.588** | Naive bayes | **0.333** | 0.25 | 0.3 |
|  | Support Vector Machine | 0.21 | **0.538** | 0.118 | Random forest | **0.454** | **0.454** | **0.333** | Logistic regression | **0.333** | 0.32 | 0.235 |
|  | Decision tree | 0.222 | 0.308 | **0.444** | Naive bayes | 0.286 | **0.609** | **0.4** | Decision tree | 0.316 | 0.24 | **0.375** |
|  | Random forest | 0.3 | **0.417** | **0.444** | Adaboost | **0.5** | **0.56** | **0.381** | Three-layer Perceptron | 0.118 | **0.48** | **0.333** |
| **Extraversion** | Naive bayes | 0.19 | 0 | **0.37** | Three-layer Perceptron | 0.105 | **0.4** | 0.174 | Naive bayes | 0.286 | 0 | **0.385** |
|  | Support Vector Machine | 0.1 | 0.273 | 0.2 | K-nearest neighbour | 0.273 | **0.435** | 0 | Random forest | 0 | **0.375** | 0.308 |
|  | Logistic regression | 0.273 | **0.364** | 0.111 | Logistic regression | **0.381** | **0.364** | 0.21 | K-nearest neighbour | 0.133 | **0.333** | 0.286 |
|  | K-nearest neighbour | 0.111 | **0.381** | 0.261 | Random forest | **0.348** | 0.3 | 0.316 | Three-layer Perceptron | 0 | **0.375** | **0.385** |
|  | Three-layer Perceptron | 0.111 | 0.3 | **0.417** | Naive bayes | **0.4** | 0.286 | **0.348** | Support Vector Machine | 0.118 | **0.348** | 0.3 |
|  | Decision tree | **0.435** | 0.3 | 0.316 | Decision tree | **0.385** | **0.353** | **0.421** | Logistic regression | 0.105 | **0.4** | 0.286 |
|  | Random forest | 0.32 | **0.375** | **0.381** | Support Vector Machine | **0.5** | **0.476** | **0.353** | Adaboost | 0.21 | **0.444** | **0.348** |
|  | Adaboost | 0.286 | **0.526** | 0.273 | Adaboost | **0.417** | **0.555** | **0.4** | Decision tree | **0.364** | 0.316 | **0.353** |
| **Openness** | Adaboost | 0.273 | 0.1 | 0.3 | Three-layer Perceptron | **0.345** | 0 | **0.353** | Random forest | 0.2 | 0.273 | 0.111 |
|  | Decision tree | 0.21 | 0.286 | 0.273 | Support Vector Machine | **0.414** | 0 | 0.316 | Decision tree | 0.316 | 0.118 | **0.417** |
|  | Random forest | 0.19 | 0.316 | **0.364** | Adaboost | **0.387** | 0.25 | 0.133 | Naive bayes | 0.222 | 0.3 | **0.454** |
|  | Support Vector Machine | 0.286 | 0.235 | **0.353** | Decision tree | 0.261 | 0.316 | 0.3 | Adaboost | 0.273 | **0.353** | **0.381** |
|  | Three-layer Perceptron | 0.24 | 0.235 | **0.4** | Random forest | 0.182 | 0.286 | **0.421** | K-nearest neighbour | **0.4** | 0.154 | **0.454** |
|  | K-nearest neighbour | **0.37** | 0.118 | **0.444** | Logistic regression | 0.296 | 0.133 | **0.5** | Three-layer Perceptron | 0.273 | 0.21 | **0.526** |
|  | Logistic regression | **0.333** | 0.3 | **0.444** | K-nearest neighbour | **0.414** | 0.143 | **0.421** | Logistic regression | 0.273 | **0.364** | **0.375** |
|  | Naive bayes | **0.333** | 0.316 | **0.48** | Naive bayes | 0.235 | **0.4** | **0.48** | Support Vector Machine | **0.348** | 0.316 | **0.444** |
| **Agreeableness** | Decision tree | 0 | 0.16 | 0.273 | Naive bayes | 0.261 | 0.095 | 0.111 | Decision tree | 0 | 0.095 | 0.091 |
|  | Logistic regression | 0.174 | 0.2 | 0.316 | Random forest | 0.2 | 0.095 | 0.19 | Random forest | 0.21 | 0.118 | **0.333** |
|  | Adaboost | 0.21 | 0.125 | **0.37** | Decision tree | 0 | 0.261 | 0.286 | K-nearest neighbour | 0.2 | 0.235 | 0.261 |
|  | Random forest | 0 | 0.2 | **0.538** | Logistic regression | 0.182 | 0.25 | 0.25 | Three-layer Perceptron | 0.2 | 0.2 | 0.3 |
|  | Naive bayes | **0.364** | **0.333** | 0.182 | Support Vector Machine | 0.222 | 0.24 | 0.316 | Logistic regression | 0.182 | **0.353** | **0.381** |
|  | Support Vector Machine | 0.222 | **0.417** | **0.4** | Three-layer Perceptron | 0.222 | 0.286 | **0.435** | Adaboost | 0.222 | 0.267 | **0.444** |
|  | Three-layer Perceptron | 0.25 | **0.417** | **0.454** | Adaboost | **0.333** | 0.316 | **0.4** | Support Vector Machine | 0.19 | **0.444** | **0.381** |
|  | K-nearest neighbour | 0.3 | **0.526** | **0.435** | K-nearest neighbour | **0.353** | **0.435** | 0.273 | Naive bayes | **0.454** | **0.381** | **0.353** |
| **Conscientiousness** | Logistic regression | 0 | 0.2 | 0.077 | Random forest | 0 | 0.167 | 0.261 | K-nearest neighbour | 0 | 0.32 | 0.25 |
|  | K-nearest neighbour | 0 | 0.286 | 0.261 | Support Vector Machine | 0 | **0.353** | 0.111 | Support Vector Machine | 0 | 0.32 | 0.261 |
|  | Support Vector Machine | 0 | 0.3 | 0.261 | K-nearest neighbour | 0 | **0.4** | 0.125 | Logistic regression | 0.154 | 0.273 | 0.24 |
|  | Adaboost | 0 | 0.3 | **0.364** | Adaboost | 0 | **0.357** | 0.286 | Three-layer Perceptron | 0.286 | 0.174 | 0.261 |
|  | Three-layer Perceptron | 0.286 | **0.33** | 0.111 | Logistic regression | 0.133 | **0.385** | 0.19 | Random forest | **0.333** | 0.095 | 0.296 |
|  | Decision tree | 0.143 | 0.231 | **0.364** | Three-layer Perceptron | 0 | **0.357** | **0.381** | Naive bayes | 0.261 | 0.273 | 0.267 |
|  | Random forest | 0.267 | 0.182 | 0.32 | Decision tree | 0.235 | **0.333** | **0.381** | Decision tree | 0.308 | 0.182 | **0.4** |
|  | Naive bayes | 0.222 | **0.461** | 0.222 | Naive bayes | **0.364** | **0.348** | **0.353** | Adaboost | 0.267 | 0.261 | **0.454** |
| **Machiavellianism** | Naive bayes | 0 | **0.345** | 0.095 | Logistic regression | 0.222 | **0.4** | 0 | Adaboost | 0.235 | **0.385** | 0.118 |
|  | Logistic regression | 0.21 | 0.32 | 0 | Random forest | **0.364** | 0.308 | 0 | Logistic regression | 0.286 | **0.348** | 0.125 |
|  | Decision tree | **0.4** | 0.261 | 0 | Adaboost | 0.25 | **0.428** | 0.222 | K-nearest neighbour | 0.286 | **0.485** | 0 |
|  | Random forest | 0.25 | **0.428** | 0.111 | Naive bayes | **0.4** | **0.592** | 0 | Naive bayes | 0.286 | **0.414** | 0.118 |
|  | Support Vector Machine | **0.4** | **0.5** | 0 | Support Vector Machine | **0.47** | **0.562** | 0 | Decision tree | **0.5** | **0.4** | 0.133 |
|  | Adaboost | 0.267 | **0.483** | 0.222 | Decision tree | **0.526** | **0.518** | 0.125 | Random forest | **0.5** | **0.4** | 0.133 |
|  | Three-layer Perceptron | **0.522** | **0.48** | 0 | K-nearest neighbour | **0.588** | **0.581** | 0.143 | Support Vector Machine | **0.444** | **0.518** | 0.133 |
|  | K-nearest neighbour | 0.308 | **0.571** | 0.143 | Three-layer Perceptron | **0.667** | **0.552** | 0.133 | Three-layer Perceptron | **0.609** | **0.5** | 0 |
| **Narcissism** | Decision tree | 0.1 | 0.286 | 0.143 | Decision tree | 0.16 | 0.21 | 0.111 | Three-layer Perceptron | 0.19 | 0.235 | 0.182 |
|  | K-nearest neighbour | 0.167 | **0.444** | 0 | Adaboost | 0.01 | 0.32 | 0.133 | Naive bayes | 0 | **0.381** | 0.32 |
|  | Logistic regression | 0.1 | **0.435** | 0.105 | Naive bayes | 0.105 | **0.467** | 0 | Adaboost | 0.231 | 0.222 | 0.25 |
|  | Adaboost | 0.21 | **0.345** | 0.143 | K-nearest neighbour | 0.111 | **0.485** | 0 | K-nearest neighbour | 0.308 | **0.455** | 0 |
|  | Random forest | 0.105 | **0.4** | 0.222 | Three-layer Perceptron | 0 | **0.667** | 0 | Logistic regression | 0.182 | **0.444** | 0.2 |
|  | Support Vector Machine | 0.308 | **0.522** | 0 | Random forest | 0.214 | **0.333** | 0.125 | Random forest | 0.235 | **0.348** | 0.3 |
|  | Naive bayes | 0.222 | **0.476** | 0.174 | Logistic regression | 0.105 | **0.583** | 0.21 | Support Vector Machine | 0.32 | **0.5** | 0.133 |
|  | Three-layer Perceptron | **0.364** | **0.381** | 0.21 | Support Vector Machine | **0.381** | **0.621** | 0 | Decision tree | 0.19 | **0.4** | **0.421** |
| **Psychopathy** | Adaboost | 0 | **0.437** | 0.095 | Three-layer Perceptron | 0 | **0.611** | 0 | Random forest | 0 | **0.467** | 0.174 |
|  | Random forest | 0 | **0.485** | 0.1 | Adaboost | 0 | **0.37** | 0.276 | Adaboost | 0 | **0.588** | 0.125 |
|  | Naive bayes | 0.167 | **0.5** | 0 | Logistic regression | 0 | **0.555** | 0.111 | Support Vector Machine | 0 | **0.611** | 0.125 |
|  | Support Vector Machine | 0 | **0.611** | 0.1 | Support Vector Machine | 0 | **0.703** | 0.105 | Naive bayes | 0.222 | **0.357** | 0.174 |
|  | Three-layer Perceptron | 0 | **0.562** | 0.261 | Random forest | 0 | **0.611** | 0.21 | Three-layer Perceptron | 0 | **0.562** | 0.2 |
|  | K-nearest neighbour | 0.25 | **0.634** | 0 | K-nearest neighbour | 0.25 | **0.684** | 0 | K-nearest neighbour | 0.25 | **0.65** | 0 |
|  | Decision tree | 0 | **0.647** | 0.3 | Decision tree | 0.182 | **0.687** | 0.105 | Decision tree | 0.222 | **0.533** | 0.19 |
|  | Logistic regression | 0.167 | **0.516** | 0.316 | Naive bayes | **0.461** | **0.588** | 0.133 | Logistic regression | 0.154 | **0.621** | **0.333** |
| Note: the predictions above the chance level of 33% are in bold. | | | | | | | | | | | | |
